# Best Combination of Vegetable By-Products for the Shelf-Life Extension of Fresh Pasta

**DOI:** 10.3390/foods13010044

**Published:** 2023-12-21

**Authors:** Adriana Lordi, Olimpia Panza, Amalia Conte, Matteo Alessandro Del Nobile

**Affiliations:** Department of Agricultural Sciences, Food and Environment, University of Foggia, Via Napoli, 25-71122 Foggia, Italy; adriana.lordi@unifg.it (A.L.); olimpia.panza@unifg.it (O.P.); matteo.delnobile@unifg.it (M.A.D.N.)

**Keywords:** fresh pasta, shelf life, pomegranate peels, olive oil by-products, broccoli by-products

## Abstract

A combination of by-products was studied in fresh handmade pasta. Pomegranate peels and olive oil by-products were used in the range 0–6% (*w*/*w*) and properly combined in a total of nine combinations with an equal amount of broccoli by-products (10% *w*/*w*). The broccoli by-products were added to improve the sensory acceptance, which was compromised when the two above by-products were added to the dough. To verify the synergic effects, among these by-products, on tagliatelle shelf life, microbiological quality based on the main spoilage groups, sensory properties, appearance of visible molds, pH and moisture content were monitored in all the packaged samples stored at 4 °C. In addition to fortified pasta samples, control tagliatelle was also investigated. A mathematical approach was used to fit experimental data and calculate pasta shelf life. In addition, a mathematical model was also proposed to describe the dependence of the shelf life from each by-product percentage added to the formulation. Results showed that while the control fresh pasta lasted about 3 days for the undesired proliferation of yeasts and coliforms, all fortified samples maintained acceptable quality for at least one week. Depending on the by-product combination, shelf-life values could reach more than 13 days. The best combination of by-products calculated based on the mathematical model, that reached the highest shelf life (13.30 days), corresponded to 10% broccoli by-products combined with 6% olive oil by-products and 6% pomegranate peels.

## 1. Introduction

Every year, large quantities of food by-products are produced all over the world, thus causing impacts on the environment and human health, in addition to large costs for their disposal [1]. Consequently, the need to reduce by-products along the food chain arises, trying to prevent, where possible, their generation or to proceed towards their valorization, with a view of a circular economy [2]. There have been several attempts to valorize the food by-products at the level of scientific research. One of the most widespread approaches is their inclusion in the food formulations to improve the nutritional level [3,4,5,6], being by-products that are rich sources of macronutrients, micronutrients and phytochemicals [7].

Broccoli by-products (*Brassica oleracea*) have been defined as therapeutic raw materials [8] with recognized beneficial properties. Bioactive compounds, such as glucosinates, isothiocyanates, flavonoids, phenolic acids and tannins [9] and vitamin A, C, E, and K and several important minerals, have been found, specifically in roots and leaves [10,11]. Pomegranate (*Punica granatum* L.) peels are abundant by-products from fruit processing and, due to their well-known properties, they can be advantageously recycled [12]. Many studies in the literature show that they turned out to be powerful antimicrobial and antioxidant sources of tannins and anthocyanins [13]; moreover, they have high scavenging properties of free radicals and anti-mutagenic properties [14]. Pomegranate has been regarded as a “healing food” with numerous beneficial effects due to its important proximate composition, mineral content and antibacterial, antiviral, antidiabetic and antifungal activity [15,16]. Olive oil pomace was found to be rich in phenolic compounds [17], thus justifying its potential application to the food industry [18,19]. Different publications assessed the quality of olive oil pomace in pasta, thus demonstrating the increase in nutritional properties after the incorporation of these by-products in the food formulation [20,21,22]. 

Among the fortified food, pasta is the most preferred item because it is very simple to be prepared, it has good sensory properties and is recognized as a staple food [23]. Although pasta fortified with by-products has been abundantly studied and various types of recovered powders were explored in the last years to investigate the correlation among technological features, chemical characteristics, and sensory properties [24,25,26,27,28,29,30,31,32,33,34,35], little research is reported in the literature to verify the ability of by-products to prolong fresh pasta shelf life. Information is available on extracts from broccoli by-products [10] or pomegranate peel powder, both applied to fresh ravioli [14], which are a special type of filled pasta. In the same view, Panza, Conte and Del Nobile [36] carried out a study where fig peels enhanced the microbial and sensory quality of handmade fresh tagliatelle. 

Considering the literature on the topic, the combination of by-products to extend fresh pasta shelf life, represents a novelty. No shelf-life test was found with the aim to study the combination of different by-products in the same food formulation, to verify possible synergic interactions among the bioactive compounds and identify their best combination. Therefore, the aim of this study was to combine three different by-products (broccoli by-products, pomegranate peels and olive oil pomace) in the form of powder with fresh homemade tagliatelle. To assess their effects, nine different combinations were studied. Both the microbiological and sensory quality of pasta samples were monitored for a proper period of storage. The values of pH and moisture content were also monitored. Contents of phenols and flavonoids, as well as the antioxidant activity, of both fortified and unfortified samples were assessed. Comparisons among by-product effects on pasta shelf life were evaluated by using a mathematical approach.

## 2. Materials and Methods

### 2.1. Raw Materials

Three different by-products from broccoli roots and leaves (50%), pomegranate peels and olive oil pomace were properly combined and added to a fresh handmade tagliatelle formulation. A local farm located in Foggia (Italy) kindly provided broccoli by-products (*Brassica oleracea*). A dryer (SG600, Namad, Rome, Italy) at 35 °C for 48 h was used for by-product dehydration. A local horticultural association (A.P.O. Foggia, Italy) kindly provided pomegranate fruits (*Punica granatum*, cv. Wonderful). Pomegranates were carefully washed in water, then immersed for 1 min in chlorinated water (20 mL L^−1^) and rinsed again in tap water. Pomegranate peel and pulp were separated and dried in a food dehydrator (Melchioni-Babele, Milan, Italy) at 50 °C for 48 h. The olive oil pomace from the Cellina di Nardò cultivar was obtained in an olive mill located in Bisceglie (Bari, Italy) by using a two-phase decanter called the Pieralisi Leopard system. The milling process combines modern extraction technology without water addition and recovers a certain quantity of by-products made up of wet pulp without any traces of kernel. A dehydration process of olive pomace was carried out in a dryer (SG600, Namad, Rome, Italy) at 35 °C for 72 h. All the dried powders were reduced to a fine size (<500 µm) using a hammer mill (16/BV-Beccaria s.r.l., Cuneo, Italy) and then stored under refrigerated conditions until further utilization. 

### 2.2. Tagliatelle Preparation

Three ingredients were used to produce fresh pasta: durum wheat semolina (710 g), distilled water (160 g) and fresh pasteurized eggs (130 g). Semolina and eggs were purchased from a local market (Foggia, Italy). The ingredients were mixed in a bowl (Multichef, Ariete, Firenze, Italy) for 5 min. Then, the dough was rolled out through a sheeter machine (Lineapasta Equipments, Padova, Italy) in several passages, progressively reducing the distance between the rollers, to obtain a sheet of 2 mm. Finally, the tagliatelle with a width of 6 mm and a length of 20 cm was obtained by using the cut of the same machine (Lineapasta Equipments, Padova, Italy). Different formulations were prepared: pasta without any by-product addition (Ctrl#1 and Ctrl#2), and nine types of pasta with different combinations of by-products. A concentration equal to 10% (*w*/*w*) of broccoli by-products was maintained in all the doughs. Olive oil by-products and pomegranate peels ranged from 0 to 6% according to the nine combinations reported in Table 1. Due to the numerosity of samples, two steps were carried out, the first with Ctrl#1 and five samples, 0%BOP-0%PMG, 3%BOP-0%PMG, 6%BOP-0%PMG, 0%BOP-3%PMG and 3%BOP-3%PMG, and the other one with Ctrl#2 and the last four samples, 6%BOP-3%PMG, 0%BOP-6%PMG, 3%BOP-6%PMG and 6%BOP-6%PMG. The water used to hydrate the powders was different, depending on the type and amount of by-product. Details are reported in the last column of Table 1. 

An amount of 100 g of each sample in each step was packaged in a polystyrene tray (200 × 100 mm) and then closed in air using a high-barrier bag (250 × 350 mm, thickness 100 μm, Biochemia, Bari, Italy). All the fresh pasta samples were kept under refrigeration (4 ± 1 °C) for a storage period of 1 month.

### 2.3. Microbiological Quality of Fresh Tagliatelle

#### 2.3.1. Microbiological Analyses

Microbiological analyses were conducted to count spoilage bacteria, molds, and yeasts during the storage period. About 10 g of fresh tagliatelle was diluted with NaCl solution (9 g/L) and homogenized. Subsequently, serial dilutions were plated in Petri dishes. For mesophilic and psychrotrophic bacteria, Plate Count Agar (PCA, Oxoid) incubated at 30 °C for 48 h and 4 °C for 10 d was used, respectively; for coliforms, Violet Red Bile Lactose Agar (VRBA) incubated at 37 °C for 24 h was used; Sabouraud Dextrose Agar, with 0.1 g/chloramphenicol added (C. Erba, Milan, Italy), incubated at 25 °C for 48 h, was used for yeasts and at 25 °C for 5 d for molds; and Baird-Parker Agar, supplemented with egg yolk tellurite emulsion, incubated at 37 °C for 48 h, was used for Staphylococcus spp. At each sampling time, two different samples were used for test repetition. Microbial thresholds were set to 10^6^ cfu/g for total viable mesophilic and psychrotrophic bacteria and 10^4^ cfu/g for Staphylococcus spp., coliforms, yeasts, and molds (Reg. CE n. 2073/2005). In addition to counted molds, the presence of visible molds on the product surface was also detected, and the first day when it appeared was indicated as an additional index for defining the product shelf life. 

#### 2.3.2. Microbiological Data Modeling

A simple model is proposed in this study to quantitatively describe the microbial growth and determine the time at which the viable cell concentration reached its threshold (i.e., the Microbial Acceptability Limit—MAL). The model is based on two hypotheses: (a) the growth kinetic follows a first order kinetic-type equation with a time-dependent kinetic constant; (b) ${\forall \;t\;\leq \;t}_{lag}\;\to \;\frac{dN\left(t\right)}{dt}=0$; ${\forall \;t>t}_{lag}\;\to \;\frac{dN\left(t\right)}{dt}>0$, where $N\left(t\right)$ is the viable cell concentration at the time t, and $t_{lag}$ is the lag time. 

The following equation is proposed to describe the time course of the viable cell concentration ${\forall \;t>t}_{lag}$:

(1) $$\frac{dN\left(t\right)}{dt}=\frac{K_{1}\cdot \left(t-t_{lag}\right)}{1+{\left(t-t_{lag}\right)}^{K_{2}}}\cdot N\left(t\right)$$

Which can be rearranged as:

(2) $$\frac{dln\left(N\left(t\right)\right)}{dt}=\frac{K_{1}\cdot \left(t-t_{lag}\right)}{1+{\left(t-t_{lag}\right)}^{K_{2}}}$$

where $K_{i}$ are the model parameters, which are assumed to be constant. It is worth noting that the term $\frac{K_{1}\cdot \left(t-t_{lag}\right)}{1+{\left(t-t_{lag}\right)}^{K_{2}}}$ can be interpreted both as the time-dependent kinetic constant and as the specific growth rate. Considering that the time course of the viable cell concentration is generally expressed as $log\left(N\left(t\right)\right)\;vs\;time$, the proposed model can be rewritten as follows:

(3) $$\frac{d\left(logN\left(t\right)\right)}{dt}=\frac{K_{1}^{\prime }\cdot \left(t-t_{lag}\right)}{1+{\left(t-t_{lag}\right)}^{K_{2}}}$$

where $K_{1}^{\prime }=\frac{K_{1}}{ln\left(10\right)}$.

### 2.4. Sensory Quality of Fresh Tagliatelle

#### 2.4.1. Sensory Analyses

The handmade pasta samples in both trials were subjected to a sensory evaluation. The panel test was composed by seven trained panelists (females, age between 28 and 48 years), Researchers of the Food Department with high experience in evaluating fresh pasta before the current study. A quantitative descriptive analysis (QDA) was used to evaluate the intensity of each sensory attribute. The panelists were asked to indicate the following attributes of both cooked and uncooked samples: odor, color, homogeneity, and resistance to breaking of fresh uncooked tagliatelle and odor, color, elasticity, and firmness of fresh cooked pasta. In addition, each panel member was asked to give the global sensory score of cooked and uncooked samples. A 9-point scale was used for the assessment of each specific sensory attribute and of the global sensory quality (1 = lowest score; 9 = highest score). The score equal to 5 was taken as the threshold for acceptability. This experiment did not require Ethics Committee approval because there were no risks associated for panelists who judged the samples. The sensory evaluation was carried out on small samples of raw and cooked pasta prepared with food-grade ingredients and according to good manufacturing practices. The panelists, as Researchers of the University of Foggia, were fully informed of the research conducted, and they knew how their data would be used.

#### 2.4.2. Sensory Data Modeling

The evolution, during storage, of the overall quality of the investigated samples was described using the following decreasing sigmoid:

(4) $$OQ=A_{1}-A_{2}\cdot exp\left\{-exp\left[\left(A_{3}\cdot 2.7182\cdot \frac{A_{4}-t}{A_{2}}\right)\right]+1\right\}$$

where OQ is the overall quality, t is the time and $A_{i}$ are the model’s parameters. In fact, Equation (4) was obtained by adapting the Gompertz function as modified by Zwietering, Jougerburger, Rombouts and van’T Riet [37]. 

### 2.5. Chemical Quality of Fresh Tagliatelle

#### 2.5.1. Reagents

The following chemicals were adopted for the analyses: Folin–Ciocalteu reagent, gallic acid monohydrate, anhydrous sodium carbonate, methanol, hydrochloric acid, 2,2-azino-bis (3-ethylbenzothiazoline-6- sulfonic acid) diammonium salt (ABTS), potassium persulfate, Trolox (6-hydroxy-2,5,7,8- tetramethylchroman-2-carboxylic acid), aluminum chloride, sodium nitrite, sodium hydroxide solution and quercetin, supplied by Sigma-Aldrich (Milan, Italy). All reagents were of analytical grade. 

#### 2.5.2. Extraction from Fresh Tagliatelle

To determine total phenols (TPCs), flavonoids (TFCs) and antioxidant activity (ABTS), the extraction of all fresh tagliatelle samples was performed as described by Panza et al. [36] with slight modifications. Initially, all the samples were dried in a ventilated stove (BINDER GmbH, Tuttlingen, Germany) at 35 °C and milled to obtain a powder. Two grams of dried samples was mixed with 20 mL of acidified methanol (80% MeOH in H_2_O acidified with 1% HCl). The mixtures were shaken at room temperature in the dark for 2 h at 3000 rpm (HS 260 BASIC, IKA, Staufen, Germany) and centrifuged at 5 °C for 15 min at 10,000 rpm (5804R, Eppendorf, Milan, Italy). Then, the supernatant was collected and filtered (PTFE, 0.45 µm) prior to the analytical determinations. All the extractions were made in triplicate, with appropriate dilutions. 

#### 2.5.3. Total Phenolic Content

The Folin–Ciocalteu method, as described by Panza et al. [36], was used for the total phenolic content (TPC). TPC was expressed as milligrams of gallic acid equivalents (GAE)/gram of dry weight (dw), according to a calibration curve (3.12–100 mg/L; R2 = 0.999). 

#### 2.5.4. Total Flavonoid Content

The aluminum chloride colorimetric method, as described by Panza et al. [36], was used for the total flavonoid content (TFC). TFC was expressed in milligrams of quercetin equivalent (QE) per gram of dry weight (dw), according to a calibration curve (6.25–400 mg/L; R2 = 0.995). 

#### 2.5.5. Antioxidant Activity

The ABTS method, as described by Panza et al. [36], was used for the antioxidant activity of fresh tagliatelle. The antioxidant activity was expressed as milligrams of Trolox equivalents/gram of dry weight (dw), according to a calibration curve at concentrations between 12.5 and 500 mg/L (R2 = 0.990). All analyses were carried out in triplicate.

### 2.6. Moisture Content

Moisture content was determined using a thermal balance (Sartorius, Gottingen, Germany). Five grams of sample was uniformly distributed on an aluminum plate and placed in the thermal balance set at 130 °C. For each sample, two replicates were measured. 

### 2.7. pH Measurement

The pH measurement was performed on the first homogenized dilution of each sample, using a pH meter (Crison, Barcelona, Spain). For the repetition, two samples were used at each sampling time.

### 2.8. Pasta Shelf Life

The following mathematical model has been proposed to describe the dependence of the shelf life (SL) on the percentage of by-products from BOP and PMG added to tagliatelle:

(5) $$SL=\left(a_{1}+b_{1}\cdot \%BOP\right)+\left(a_{2}+b_{2}\cdot \%BOP\right)\cdot {\left(\%PMG\right)}^{4}$$

where %BOP and %PMG are the BOP and PMG percentage added to fresh pasta, respectively; they are the two independent variables, whereas SL is the dependent variable, and $a_{i}$ and $b_{i}$ are the model’s parameters.

To quantitatively assess the ability of Equation (5) to fit the experimental data, the relative percent difference ($\bar{E}\%$) was used [38], and it is defined as follows:

(6) $$\bar{E}\%=\frac{100}{N}\cdot \sum_{i=1}^{i=N}\frac{\left|M_{exp}-M_{pred}\right|}{M_{exp}}$$

where N is the number of experimental points, $M_{exp}$ is the experimental value and $M_{pred}$ is the predicted value.

### 2.9. Statistical Analysis

One-way ANOVA analysis was used to compare the experimental data. Duncan’s multiple range test, with the option of homogeneous groups (*p* < 0.05), was used to determine significance among differences. To this aim, Statistica 7.1 for Windows (StatSoft Inc., Tulsa, OK, USA) was used.

## 3. Results and Discussion

In this study, three different by-products were combined in pasta dough to identify the possible synergic effects among them and select their best combination. Specifically, pomegranate peels and olive oil pomace were both used in the range from 0 to 6% (*w*/*w*), according to nine combinations. An equal amount of broccoli by-products was added to all of them, mainly for sensory defect correction. As a fact, the 6% pomegranate peels and olive oil by-products compromised the full sensory acceptance of pasta, mainly due to undesired defects in the pasta flavor. Both by-products generally leave a bad taste in one’s mouth. Based on investigations carried out before the current study on fresh tagliatelle fortified with the same vegetable by-products separately used [39], the pasta acceptability was improved by adding broccoli by-products because they can attenuate the sensory defects of peels and pomace. Therefore, an addition quantified in 10% (*w*/*w*) was used for all nine combinations tested, being enough to greatly improve the fortified pasta acceptability. 

### 3.1. Moisture Content, pH and Chemical Quality

The percentage moisture content and the pH of pasta samples were monitored for about 1 month in all the control and fortified pasta samples. Results demonstrated that the percentage moisture content remained quite constant over time in all samples. Table 2 collects data recorded at the beginning and at the end of the storage time in both fortified and unfortified samples. As can be seen, both controls (Ctrl#1 and Ctrl#2) recorded a moisture content of around 31%. Fortified samples of the combinations 0%BOP-0%PMG, 0%BOP-3%PMG, 0%BOP-6%PMG and 3%BOP-0%PMG recorded a moisture content of about 35%; the other five combinations recorded a moisture content of around 37%. These slight differences among samples could most probably be due to the more abundant amount of water added to the dough during pasta production to hydrate the by-products, as detailed in the previous Table 1. Similar experimental findings in terms of moisture content have also been reported in other studies where the same fruit and vegetable by-products were added to a fresh handmade pasta formulation [36,39].

In terms of pH, a small decline appeared over time in all the samples investigated. The differences became statistically significant (*p* > 0.05) when the beginning and the end of the observation period were compared (Table 3). Paying attention to both fortified and unfortified pasta samples, the differences in terms of pH were more marked (*p* < 0.05). This evidence is not surprising because generally the addition of by-products to food formulations and, in particular, pomegranate peel addition, provokes a pH reduction, as the peel pH is around 5 [40]. 

Data in Table 4 show the content in polyphenols and flavonoids recorded in all the pasta samples. It is worth noting that, if compared to the other fortified products, the control pasta recorded lower values of bioactive compounds, thus demonstrating that the addition of by-products greatly improved the nutritional content of fresh handmade tagliatelle. The most striking feature of Table 4 is the importance of pomegranate peel addition, because its presence in the formulation greatly improved the content of both polyphenols and flavonoids. This evidence is consistent with the high content of bioactive compounds in the peels, also abundantly reviewed in the literature [41]. According to the experimental data recorded in the current research, the results of the antioxidant activity reported in the last column of the same table demonstrate the ability of by-products to improve the antioxidant properties of the final pasta. Improvements in antioxidant activity were also recorded when these by-products were adopted separately [36,39], and it is reasonable to assume that their combination further increases the nutritional content of the final product. 

### 3.2. Microbiological Quality of Handmade Fresh Tagliatelle

**Figure 1:**
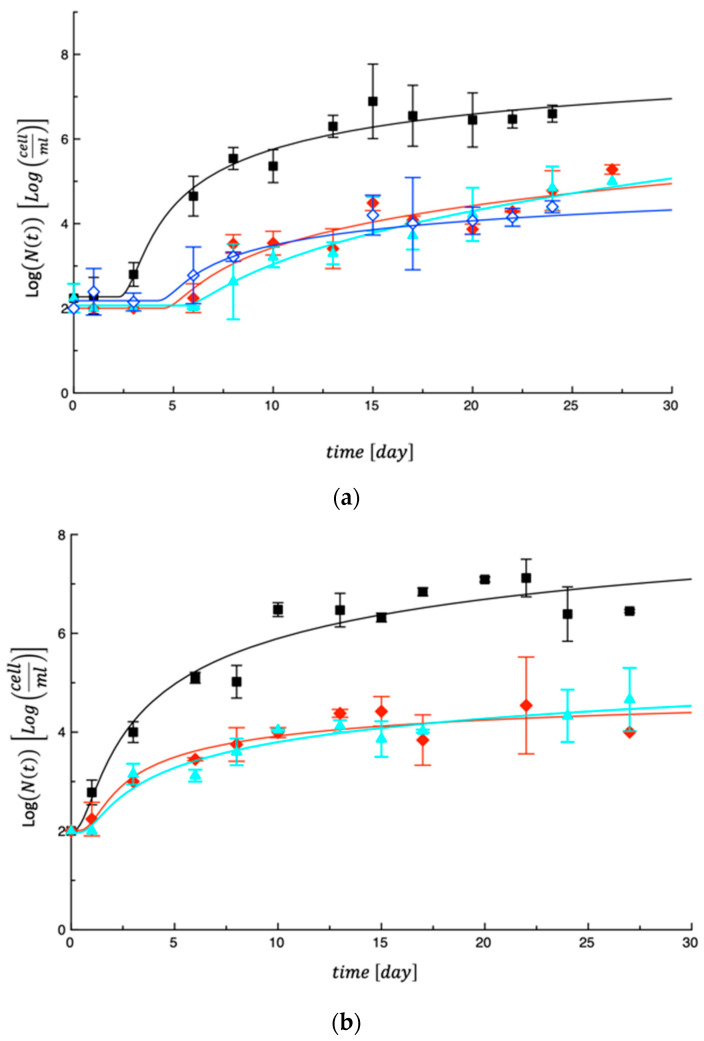
(**a**) Evolution of yeasts in samples tested in the first step. Ctrl#1 = black; Sample 6%BOP-0%PMG = red; Sample 3%BOP-0%PMG = bright blue; Sample 3%BOP-3%PMG = dark blue. The curves in the figure are the best fit of Equation (3) to the experimental data. (**b**) Evolution of yeasts in samples tested in the second step. Ctrl#2 = black; Sample 6%BOP-3%PMG = red; Sample 6%BOP-6%PMG = bright blue. The curves in the figure are the best fit of Equation (3) to the experimental data.

Fresh pasta samples were monitored for about 1 month for microbiological quality. During this time, different fungal and microbial growths were observed in tagliatelle enriched with by-products and in the corresponding control samples. Interesting differences were also found among the various fortified samples. With regards yeasts and molds, similar trends were recorded. As an example, Figure 1a shows the evolution of the yeast concentration in some of the investigated samples tested in the first step. As can be seen, yeasts in the control pasta (Ctrl#1) grew very rapidly and reached their highest concentration (around 10^7^ cfu/g) within 10 days, whereas in the samples with different combinations of by-products (0%BOP-6%PMG, 3%BOP-0%PMG, 3%BOP-3%PMG), the fungal proliferation was slowed down. No great differences were detected among the fortified samples (*p* > 0.05); all of them presented a lag phase of about 5 days and then a slow proliferation up to 10^4^ cfu/g.

Comparable results were also found in Figure 1b, where some other fortified samples tested in the second step are shown (6%BOP-3%PMG, 6%BOP-6%PMG), in comparison with the respective control pasta (Ctrl#2), which proliferated up to 10^7^ cfu/g. Also, in this case, the yeasts in the fortified samples presented low proliferation up to 10^4^ cfu/g. These interesting antifungal effects were also recorded when the same by-products were used separately in the pasta formulation and were found to be very effective when the by-product percentage was higher than 5% (*w*/*w*) [39]. Even though information about by-product combinations are not available in the literature, the recognized properties of broccoli by-products [9], pomegranate peels [16] and olive oil by-products [17] in terms of bioactive compounds with antifungal activity could support the assumption that when combined, their effects can be amplified. 

**Figure 2:**
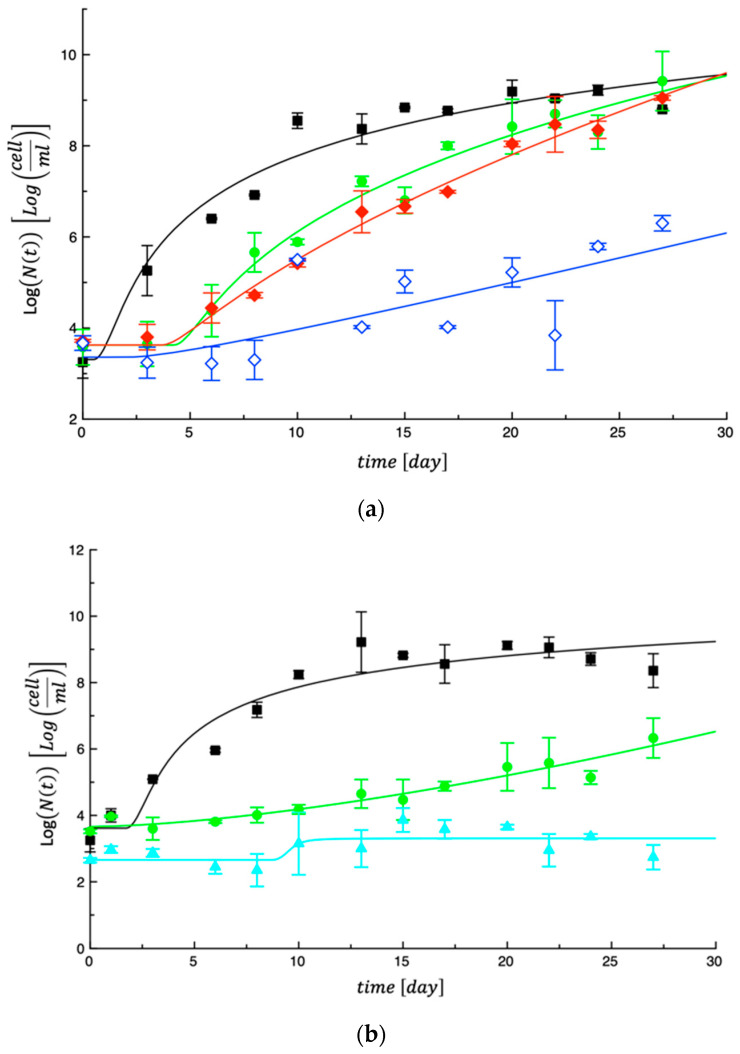
(**a**) Evolution of mesophilic bacteria in the samples tested in the first step. Ctrl#1 = black; Sample 0%BOP-3%PMG = green; Sample 0%BOP-6%PMG = red; Sample 3%BOP-3%PMG = dark blue. The curves in the figure are the best fit of Equation (3) to the experimental data. (**b**) Evolution of mesophilic bacteria in samples tested in the second step. Ctrl#2 = black; Sample 6%BOP-0%PMG = green; Sample 6%BOP-6%PMG = bright blue. The curves in the figure are the best fit of Equation (3) to the experimental data.

In terms of mesophilic and psychrotrophic bacteria, the combined by-products have not always exerted an effective antimicrobial effect. Figure 2a shows the evolution of mesophilic bacteria for the control (Ctrl#1) and three by-product combinations (0%BOP-3%PMG, 0%BOP-6%PMG, 3%BOP-3%PMG) tested in the first step. As one can observe, only the combination 3%BOP-3%PMG was effective in promoting significant microbial inhibition. In the subsequent experimental step, among the combinations tested, those related to 6%BOP-0%PMG and 6%BOP-6%PMG were found to be very interesting, if compared to the Ctrl#2 (Figure 2b). These data demonstrate that proper combinations of the three by-products are necessary to reach significant effectiveness against mesophilic bacteria. To explain these results, we can consider that when used alone, these by-products were found to be effective only when used at least in concentrations equal or higher than 5% [39], thus confirming that small percentages of fortification are not enough to control microbial proliferation. 

Most of the combinations tested in both steps were found to be effective against coliforms which generally were found to be less resistant than mesophilic and psychrotrophic bacteria [36,39]. No *Staphylococcus* spp. counts were detected in the investigated samples during the entire observation period, thus confirming the hygienic conditions adopted during pasta processing. 

The curves shown in the Figure 1 and Figure 2 were obtained by fitting Equation (3), reported in the modeling section, to the experimental data. As can be inferred from what is reported in these two figures, despite its simplicity, the proposed model interpolates the data quite satisfactorily. The model parameters obtained by fitting Equation (3) to the experimental data were used to calculate the MAL value, intended as the time at which each microbial species reached its threshold (10^6^ cfu/g for total viable mesophilic and psychrotrophic bacteria, 10^4^ cfu/g for coliforms, yeasts and molds). The obtained results are listed in Table 5. In the same Table, the day when the visible molds appeared is also indicated (VM).

As can be seen, the investigated samples recorded different MAL values, thus demonstrating that the combination of by-products exerted different antimicrobial and antifungal effects on handmade tagliatelle. It is worth noting that MAL values were always higher if compared to the relative control pasta. Some differences can be observed also comparing the two control samples. This demonstrates that variability in the contamination level of the raw materials used to make the tagliatelle needs to be considered when the data are comprehended. Another striking feature of these results is the evidence that synergic effects among by-products are recorded. As a fact, previous findings about these by-products used separately demonstrated their effects on microbial proliferation and allowed for an increase in MAL values [36,39]. The results of the current research surprisingly demonstrated that a proper by-product combination can promote the control of bacterial growth for more than 1 month. 

### 3.3. Sensory Analysis

**Figure 3:**
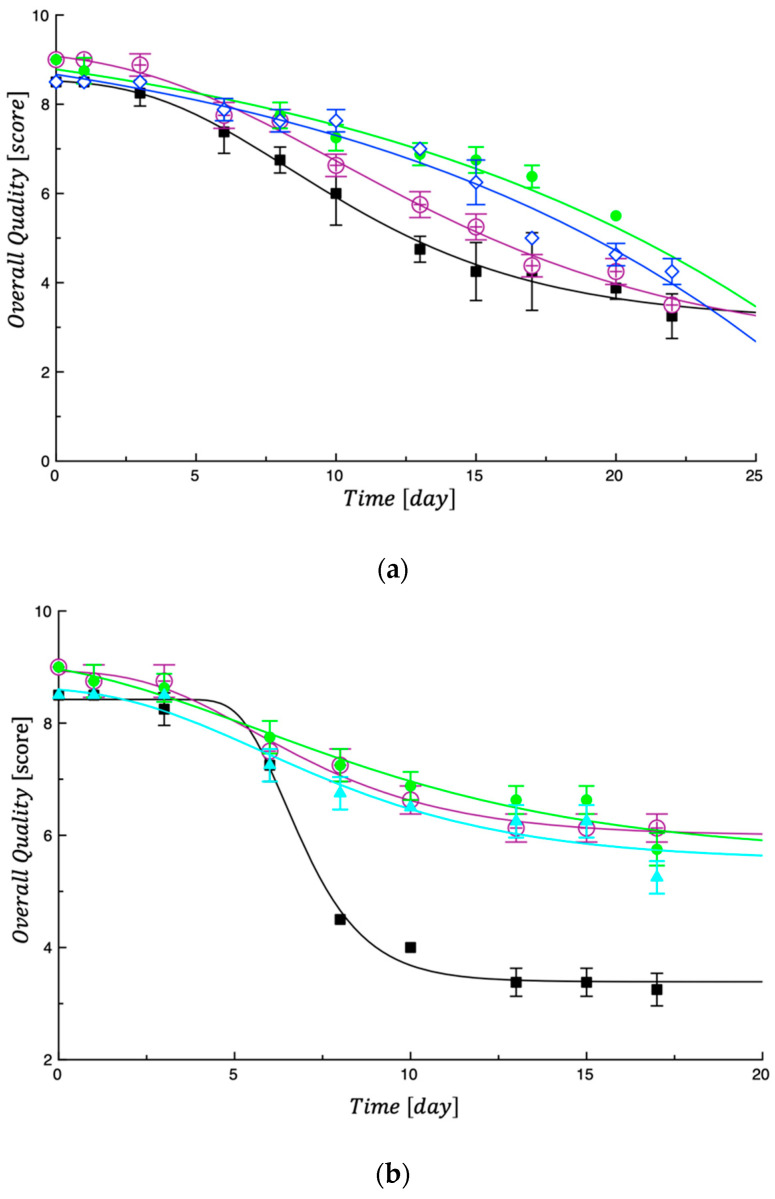
(**a**) Evolution of overall quality in cooked samples tested in the first step. Ctrl#1 = black; Sample 0%BOP-0%PMG = violet; Sample 0%BOP-3%PMG = green; Sample 3%BOP-3%PMG = dark blue. The curves in the figure are the best fit of Equation (3) to the experimental data. (**b**) Evolution of overall quality in cooked samples tested in the second step. Ctrl#2 = black; Sample 3%BOP-6%PMG = violet; Sample 6%BOP-0%PMG = green; Sample 6%BOP-6%PMG = bright blue. The curves in the figure are the best fit of Equation (4) to the experimental data.

Data in Figure 3 show trends of the overall quality of cooked samples tested in the first and second steps. Figure 3a shows the overall quality of the control (Ctrl#1) and some of the investigated combinations (0%BOP-0%PMG, 0%BOP-3%PMG, 3%BOP-3%PMG) tested in the first step, and Figure 3b shows the control (Ctrl#2) and some other combinations (3%BOP-6%PMG, 6%BOP-0%PMG, 6%BOP-6%PMG). In both figures, it is possible to see that both control pasta samples became unacceptable before the investigated fortified tagliatelle, thus demonstrating that the presence of by-products in the formulations promoted a slower sensory decay during storage. This result is not new in the literature because in other research studies [10,14] where microbial and fungal proliferation was controlled by bioactive compounds addition, a better sensory quality was also recorded. 

**Figure 4:**
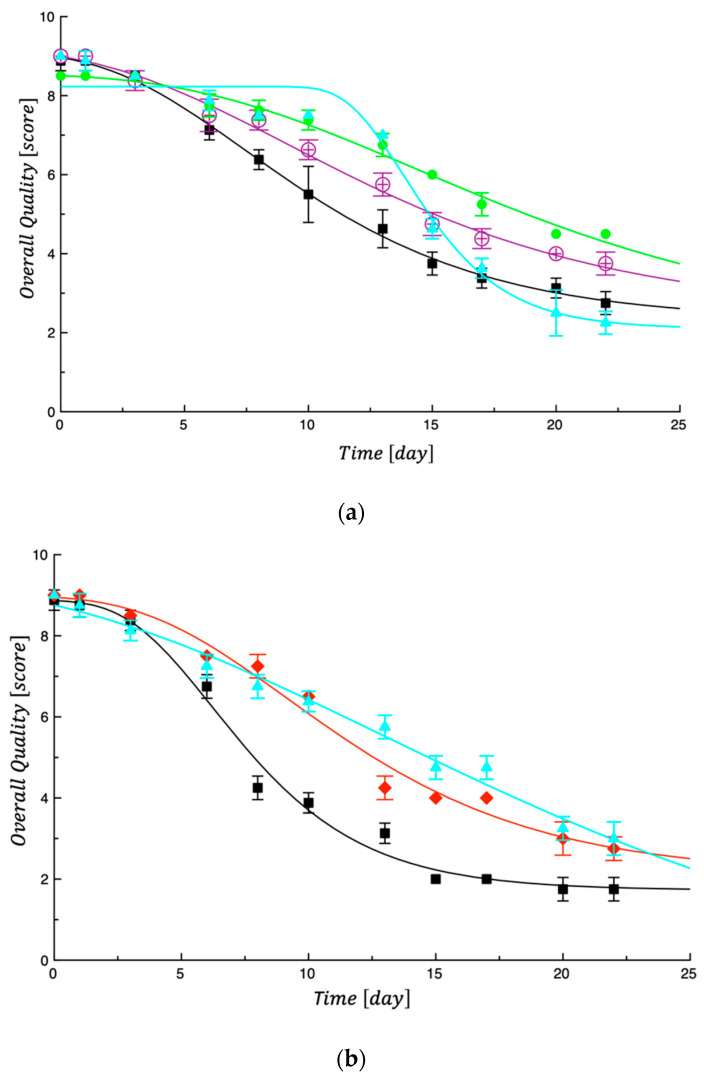
(**a**) Evolution of overall quality of uncooked samples tested in the first step. Ctrl#1 = black; Sample 0%BOP-0%PMG = violet; Sample 0%BOP-3%PMG = green; Sample 3%BOP-0%PMG = bright blue. The curves in the figure are the best fit of Equation (3) to the experimental data. (**b**) Evolution of overall quality in uncooked samples tested in the second step. Ctrl#2 = black; Sample 6%BOP-3%PMG = red; Sample 6%BOP-6%PMG = bright blue. The curves in the figure are the best fit of Equation (4) to the experimental data.

From the sensory point of view, it is worth considering that the differences among control and fortified samples were less marked when uncooked pasta was taken into account. As a fact, Figure 4 shows the data of uncooked tagliatelle in the first and second steps. 

The curves shown in Figure 3 and Figure 4 were obtained by fitting Equation (4), reported in the modeling section of sensory data. As can be observed, Equation (4) adequately interpolates the experimental data. As performed previously for the calculation of the MAL values, Equation (4) was also used in this case to calculate the SAL, intended as the time at which the overall quality reached its threshold (Score = 5). The $A_{i}$ values used were those obtained by fitting the experimental data. To comprehend the obtained SAL values (see Table 5), it must be taken into account that the presence of the investigated by-products not only affects the rate at which fresh tagliatelle overall quality decays during storage, but it also affects the initial sensory quality of the investigated product [39]. 

What emerged from the data listed in Table 5 is that the SAL values of the fortified samples are always greater than those of the corresponding control samples, thus indicating that the investigated by-products are effective in extending the sensory quality of fresh tagliatelle. When uncooked, the main attribute responsible for pasta unacceptability was the color; when cooked pasta was considered, both color and odor became the two attributes more responsible for sensory unacceptance. Other experimental findings recorded in fortified fresh pasta stored at 4 °C also demonstrated that by-product addition slowed down the evolution of sensory attributes because active compounds contributed to maintaining the characteristics of fresh product quality for more time [10,14,36,39]. 

### 3.4. Pasta Shelf Life

**Figure 5:**
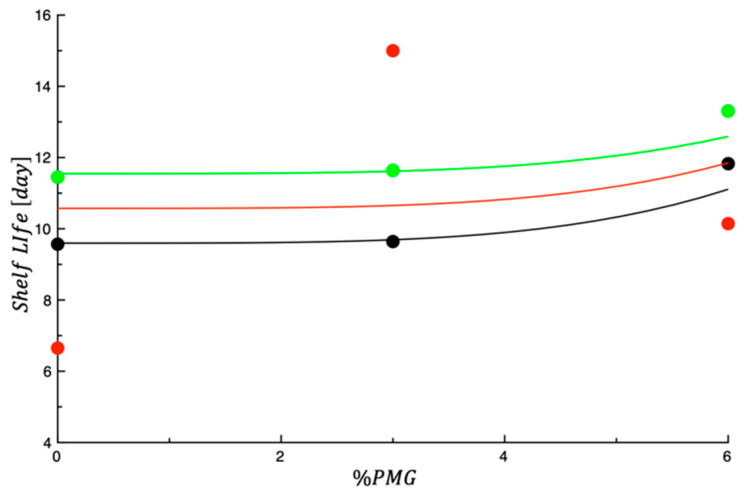
Shelf life plotted as a function of %PMG; data shown in the figure were obtained keeping %BOP = 0% (black), %BOP = 3% (red) and %BOP = 6% (green). The curves shown in the figure are the best fit of Equation (5) to the experimental data.

The pasta shelf life was obtained by taking the smallest value among the MAL and SAL of each sample and the day when the visible molds appeared, as reported in the last column of Table 5. As can be seen, the controls lasted about 3 days for the undesired proliferation of coliforms and yeasts, whereas for all the other fortified products, higher shelf-life values were recorded. The combination of by-products promoted a better maintenance of product quality with slight differences among samples. Looking at shelf life data, it is possible to infer that all the samples became unacceptable because of the high microbial proliferation and not because of detrimental phenomena ascribed to sensory decay, as also happened with previous trends reported in the literature [36,39]. As reported in the Section 2, a mathematical model has been proposed to describe the dependence of the shelf life on the percentage of by-products from BOP and PMG added to tagliatelle. Figure 5 shows the shelf life plotted as a function of %POM; each set of data shown in the figure was obtained keeping %BOP constant (i.e., %BOP = 0%; %BOP = 3%; %BOP = 6%). The curves shown in the figure are the best fit of Equation (5) reported in the Section 2, to the experimental data. It is worth noting that the fitting was performed using the entire set of data. As follows, the model parameters obtained from the fitting procedure are reported: $a_{1}=9.60\;day;\;b_{1}=0.326\;day;\;a_{2}=0.00117\;day;\;b_{2}=-6.05\cdot 10^{-5}\;day$. 

In the case of data relative to 0%BOP shown in Figure 5 (black curve), $\bar{E}\%$ equal to 13.1 was obtained. Considering the simplicity of the model used to fit the data, the $\bar{E}\%$ obtained can be considered acceptable. As can be easily inferred from the data relative to 3%BOP shown in the same Figure 5 (red curve), the inaccuracy of the fitting is largely due to the subset of data obtained when BOP was used at 3% (*w*/*w*). The inaccuracy of this specific subset of data is most probably related to the fact that two samples were tested in Step 1 (i.e., samples 3%BOP-0%PMG and 3%BOP-3%PMG) and the other in Step 2 (i.e., sample 3%BOP-6%PMG). In fact, as reported beforehand, due to the different level of microbial contamination of the raw materials used to make the tagliatelle, there is a little inconsistency between data from Step 1 and data from Step 2, as also highlighted by the two different shelf-life values obtained for the two control samples (see CTRL#1 and CTRL#2 in Table 5). The above-mentioned discrepancy between data from Step 1 and data from Step 2 may be in part responsible for the $\bar{E}\%$ value obtained in the current study. Regardless, results on pasta shelf life plotted as a function of %PMG demonstrate that a correlation between the by-product concentration and product storability can be highlighted. 

**Figure 6:**
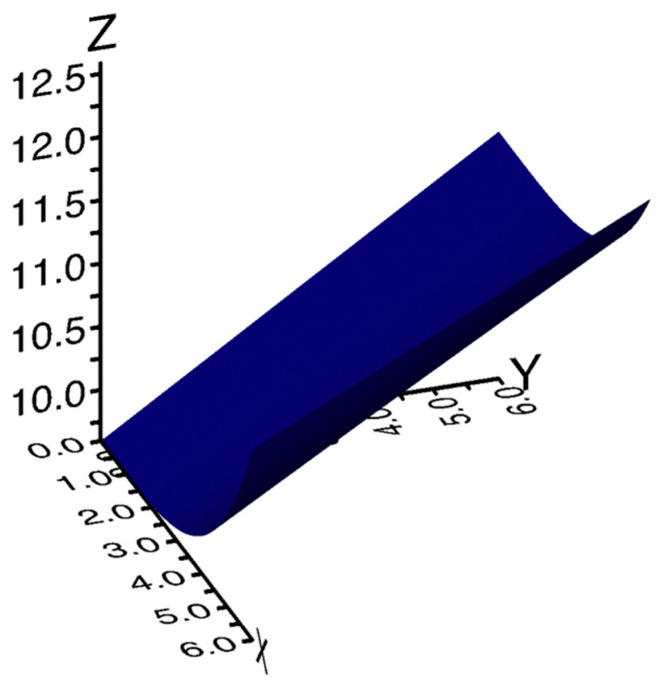
3D plot of the shelf life (*Z*-axis) vs. %PMG (*X*-axis) and %BOP (*Y*-axis). The surface plotted in the figure was obtained using Equation (5) and parameters obtained by fitting the data.

Figure 6 shows the 3D plot of the shelf life (*Z*-axis) vs. %PMG (*X*-axis) and %BOP (*Y*-axis). The surface plotted in the figure was obtained using Equation (5) and the model parameters obtained by fitting the experimental data. As can be inferred from this last figure, the shelf life steadily increased with both the %PMG and %BOP. However, a slight reduction in the slope with which the shelf life increased with the %BOP at fixed values of %PMG is observed as the %PMG increased. Data shown in the figure suggest that the best combination of the investigated vegetable residues is that with the highest concentration of both by-products, i.e., both peels and pomace used at 6% (*w*/*w*). What has been observed is not trivial. In fact, it is known that by increasing the concentration of by-products, the microbiological stability of the food is increased, and as reported in Table 5, increasing values of MAL were recorded. However, it is also observed that an increase in the by-product percentage leads to unacceptability of the food caused by a worsening of its sensory quality. In the present research, an increase in shelf life was observed with an increase in the concentration of both BOP and PMG, also considering the presence of broccoli by-products that attenuated the negative effects of PMG and BOP on food sensory quality.

## 4. Conclusions

In the current study, for the first time, more than one by-product was used in the same formulation to promote product quality preservation. Pomegranate peels and olive oil pomace were added to the dough of fresh handmade tagliatelle in different concentrations (0%, 3% and 6%, *w*/*w*), according to nine different combinations. In each pasta formulation, the same amount of broccoli by-products equal to 10% (*w*/*w*) was also added to weaken the sensory defects provoked by the addition of olive oil by-products and fruit peels. Results from the shelf-life test demonstrated that all the combinations were useful in promoting the shelf-life prolongation of fresh tagliatelle, compared to the control samples. Fresh tagliatelle without any addition lasted about 3 days, whereas all the samples with by-products recorded a longer time of storability. The mathematical approach proposed in the current study allowed for the dependence of shelf life on the percentage of pomegranate peels, when olive oil by-products were changed from 0 to 6%, to be underlined. Results are interesting, even if the fitting does not seem to be accurate for all of the data. This is probably due to a discrepancy between experimental data recorded in the two steps of our experimental plan. Apart from this weakness of our findings, it is worth noting from the results that the shelf life steadily increased with both by-products and the best combination is that with 6% BOP and 6% PMG, when 10% of broccoli by-products was also added. Experimental findings demonstrated synergic effects among by-products and allowed for the importance of the type and percentage of by-products in preserving fresh pasta to be suggested. Further research is still necessary to better assess the influence of raw material quality on product storability. Moreover, due to the increasing attention of current research to more sustainable perspectives, combinations of different by-products could also be explored for a deeper understanding of the potential of residues from fruit and vegetable processing to extend food shelf life.

## Figures and Tables

**Table 1:** Combinations of by-products added to pasta dough and amount of water to hydrate by-products.

Samples	Broccoli (%)	Olive Oil by-Products (%)	Pomegranate (%)	Water for Powder Hydration (g)
Ctrl #1	0	0	0	-
0%BOP-0%PMG	10	0	0	92.81
3%BOP-0%PMG	10	3	0	118.3
6%BOP-0%PMG	10	6	0	142.82
0%BOP-3%PMG	10	0	3	118.3
3%BOP-3%PMG	10	3	3	142.82
Ctrl #2	0	0	0	-
6%BOP-3%PMG	10	6	3	166.41
0%BOP-6%PMG	10	0	6	142.82
3%BOP-6%PMG	10	3	6	166.41
6%BOP-6%PMG	10	6	6	189.13

**Table 2:** Initial (t_i_) and final (t_f_) values of moisture content (%) of each pasta sample.

Samples	Moisture Content [t_i_]	Moisture Content [t_f_]
Ctrl #1	32.59 ± 0.01 ^G,a^	30.66 ± 0.52 ^E,b^
0%BOP-0%PMG	35.15 ± 0.06 ^F,a^	34.74 ± 1.05 ^C,a^
3%BOP-0%PMG	35.50 ± 0.43 ^F,a^	35.29 ± 0.06 ^C,a^
6%BOP-0%PMG	36.58 ± 0.04 ^C,b^	38.18 ± 0.28 ^A,a^
0%BOP-3%PMG	35.68 ± 0.18 ^E,a^	35.06 ± 0.02 ^C,b^
3%BOP-3%PMG	37.96 ± 0.08 ^A,a^	37.80 ± 0.23 ^A,a^
Ctrl #2	31.61 ± 0.10 ^H,a^	31.58 ± 0.08 ^D,b^
6%BOP-3%PMG	37.12 ± 0.13 ^B,a^	37.07 ± 0.03 ^B,b^
0%BOP-6%PMG	36.28 ± 0.08 ^D,a^	36.44 ± 0.42 ^B,a^
3%BOP-6%PMG	37.03 ± 0.03 ^B,a^	36.48 ± 0.09 ^B,b^
6%BOP-6%PMG	38.06 ± 0.06 ^A,b^	38.23 ± 0.12 ^A,a^

Data are presented as the means ± standard deviation. Data in each column with different superscript uppercase letters are statistically different (*p* < 0.05), and data in each row with different superscript lowercase letters are statistically different (*p* < 0.05).

**Table 3:** Initial (t_i_) and final (t_f_) values of pH of each pasta sample.

Samples	pH [t_i_]	pH [t_f_]
Ctrl #1	6.67 ± 0.00 ^A,a^	5.82 ± 0.02 ^A,b^
0%BOP-0%PMG	6.20 ± 0.01 ^C,a^	5.05 ± 0.01 ^E,b^
3%BOP-0%PMG	5.66 ± 0.00 ^E,a^	4.93 ± 0.01 ^G,b^
6%BOP-0%PMG	5.19 ± 0.01 ^I,a^	4.84 ± 0.02 ^H,b^
0%BOP-3%PMG	6.11 ± 0.01 ^D,a^	5.24 ± 0.04 ^C,b^
3%BOP-3%PMG	5.56 ± 0.01 ^G,a^	5.21 ± 0.01 ^C,D,b^
Ctrl #2	6.44 ± 0.01 ^B,a^	5.83 ± 0.00 ^A,b^
6%BOP-3%PMG	5.24 ± 0.01 ^H,a^	4.98 ± 0.01 ^E,b^
0%BOP-6%PMG	5.67 ± 0.01 ^E,a^	5.59 ± 0.01 ^B,b^
3%BOP-6%PMG	5.59 ± 0.01 ^F,a^	5.19 ± 0.03 ^D,b^
6%BOP-6%PMG	5.19 ± 0.01 ^I,a^	4.94 ± 0.04 ^F,b^

Data are presented as the means ± standard deviation. Data in each column with different superscript uppercase letters are statistically different (*p* < 0.05), and data in each row with different superscript lowercase letters are statistically different (*p* < 0.05).

**Table 4:** Total phenols (GAE/g dry weight), total flavonoids (QE/g dry weight) and antioxidant activity (mg Trolox equivalents/g dry weight) of each pasta sample.

Samples	TPC	TFC	ABTS
Ctrl #1	0.49 ± 0.15 ^f,g^	0.18 ± 0.06 ^e^	0.89 ± 0.13 ^e^
0%BOP-0%PMG	0.58 ± 0.07 ^e,f^	0.75 ± 0.25 ^a,b,c^	1.45 ± 0.15 ^d^
3%BOP-0%PMG	0.97 ± 0.11 ^a,b^	0.91 ± 0.15 ^a,b^	3.62 ± 0.43 ^b^
6%BOP-0%PMG	1.12 ± 0.03 ^a^	0.94 ± 0.25 ^a^	4.54 ± 0.07 ^a^
0%BOP-3%PMG	0.75 ± 0.09 ^c,d^	0.61 ± 0.07 ^b,c,d^	2.19 ± 0.20 ^c^
3%BOP-3%PMG	0.87 ± 0.12 ^b,c^	0.54 ± 0.13 ^c,d^	3.23 ± 0.42 ^b^
Ctrl #2	0.37 ± 0.03 ^g^	0.14 ± 0.05 ^e^	0.74 ± 0.51 ^e^
6%BOP-3%PMG	0.88 ± 0.08 ^b,c^	0.57 ± 0.13 ^c,d^	3.70 ± 0.10 ^b^
0%BOP-6%PMG	0.59 ± 0.07 ^e,f^	0.36 ± 0.08 ^d,e^	2.47 ± 0.07 ^c^
3%BOP-6%PMG	0.71 ± 0.07 ^d,e^	0.66 ± 0.05 ^a,b,c,d,^	3.67 ± 0.16 ^b^
6%BOP-6%PMG	1.07 ± 0.03 ^a^	0.80 ± 0.29 ^a,b,c^	4.56 ± 0.08 ^a^

^a–g^ Data in each column with different superscript letters indicate significant differences among samples (*p* < 0.05). TPC = total phenolic content; TFC = total flavonoid content; ABTS = 2,2-azino-bis(3-ethylbenzothiazoline-6-sulfonic acid diammonium salt).

**Table 5:** Values of the microbial acceptability limit (MAL) for each spoilage group, and values of overall sensory quality of both cooked and uncooked pasta sample (SAL), calculated by fitting the experimental data. The day when the visible molds (VM) appeared and the shelf life (SL) determined as the lowest values among MAL, SAL and vs. are also reported.

Sample	MAL MES.	MAL PSI.	MAL YEAST	MAL MOLD	MAL COLIF.	VM	SAL_Uncooked	SAL_Cooked	SL
			[day]			[day]	[day]		[day]
Ctrl #1	3.92	5.79	4.33	11.97	3.27	>27	12.69	11.33	3.27
0%BOP-0%PMG	9.57	11.77	11.68	>27	13.89	20	15.5	15.07	9.57
3%BOP-0%PMG	9.64	10.55	11.5	>27	24.65	20	20.76	18.79	9.64
6%BOP-0%PMG	11.83	13.61	14.58	18.72	>27	15	19.93	15.91	11.83
0%BOP-3%PMG	11.70	13.71	16.97	20.75	6.65	15	18.18	14.87	6.65
3%BOP-3%PMG	29.22	35.88	18.12	18.53	>27	15	19.18	15.09	15
Ctrl #2	4.21	4.35	2.62	8.31	3.80	>27	7.64	7.86	2.62
6%BOP-3%PMG	>27	32.21	10.14	18.99	>27	20	>17	16.11	10.14
0%BOP-6%PMG	26.24	26.76	11.45	24.28	>27	20	>17	17.60	11.45
3%BOP-6%PMG	33.18	>27	11.64	17.06	>27	13	14.05	12.55	11.64
6%BOP-6%PMG	>27	>27	13.30	17.34	>27	15	>17	14.72	13.30

MES = mesophilic bacteria; PSI = psychrotrophic bacteria; COLIF. = coliforms. The value > 27 means that no threshold was reached, and no molds appeared during the entire observation period.

## Data Availability

Data will be available on request.
